# Shifting institutional culture to develop climate solutions with Open Science

**DOI:** 10.1002/ece3.11341

**Published:** 2024-05-31

**Authors:** Julia Stewart Lowndes, Anna M. Holder, Emily H. Markowitz, Corey Clatterbuck, Amanda L. Bradford, Kathryn Doering, Molly H. Stevens, Stefanie Butland, Devan Burke, Sean Kross, Jeffrey W. Hollister, Christine Stawitz, Margaret C. Siple, Adyan Rios, Jessica Nicole Welch, Bai Li, Farnaz Nojavan, Alexandra Davis, Erin Steiner, Josh M. London, Ileana Fenwick, Alexis Hunzinger, Juliette Verstaen, Elizabeth Holmes, Makhan Virdi, Andrew P. Barrett, Erin Robinson

**Affiliations:** ^1^ University of California, Santa Barbara Santa Barbara California USA; ^2^ California Environmental Protection Agency Sacramento California USA; ^3^ NOAA Fisheries Alaska Fisheries Science Center Seattle Washington USA; ^4^ NOAA Fisheries Pacific Islands Fisheries Science Center Honolulu Hawaii USA; ^5^ ECS Federal LLC in support of NOAA Fisheries Office of Science and Technology Seattle Washington USA; ^6^ NOAA Fisheries Southeast Fisheries Science Center Miami Florida USA; ^7^ Openscapes Santa Barbara California USA; ^8^ Fred Hutch Cancer Center Seattle Washington USA; ^9^ United States Environmental Protection Agency Washington DC USA; ^10^ NASA Oak Ridge National Laboratory DAAC Oak Ridge Tennessee USA; ^11^ University of California, Los Angeles Los Angeles California USA; ^12^ NOAA Fisheries Northwest Fisheries Science Center Seattle Washington USA; ^13^ The University of North Carolina at Chapel Hill Chapel Hill North Carolin USA; ^14^ Adnet Systems, Inc. / NASA Goddard Earth Sciences Data and Information Services Center Greenbelt MD USA; ^15^ NASA Atmospheric Science Data Center Washington DC USA; ^16^ National Snow and Ice Data Center Boulder Colorado USA; ^17^ Metadata Game Changers Boulder Colorado USA; ^18^ Cooperative Institute for Marine and Atmospheric Research, University of Hawaii Honolulu Hawaii USA

**Keywords:** climate change, cloud computing, flywheel, growth mindset, open science, open source software, psychological safety

## Abstract

To address our climate emergency, “we must rapidly, radically reshape society”—Johnson & Wilkinson, All We Can Save. In science, reshaping requires formidable technical (cloud, coding, reproducibility) and cultural shifts (mindsets, hybrid collaboration, inclusion). We are a group of cross‐government and academic scientists that are exploring better ways of working and not being too entrenched in our bureaucracies to do better science, support colleagues, and change the culture at our organizations. We share much‐needed success stories and action for what we can all do to reshape science as part of the Open Science movement and 2023 Year of Open Science.

## Graphical abstract

To address our climate emergency, “we must rapidly, radically reshape society”—Johnson & Wilkinson, All We Can Save. In science, reshaping requires formidable technical (cloud, coding, and reproducibility) and cultural shifts (mindsets, hybrid collaboration, and inclusion). We are a group of cross‐government and academic scientists who are exploring better ways of working and not being too entrenched in our bureaucracies to do better science, support colleagues, and change the culture at our organizations, and we share much‐needed success stories and action for what we can all do to reshape science as part of the Open Science movement and 2023 Year of Open Science.

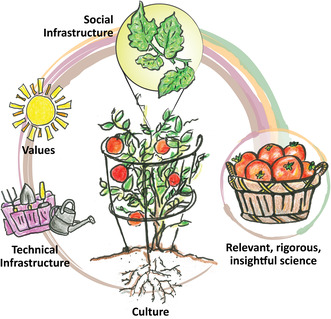

“To address our climate emergency, we must rapidly, radically reshape society. We need every solution and every solver.”‐ Ayana Elizabeth Johnson & Katharine Wilkinson, *All We Can Save*

This call to action by Drs. Johnson and Wilkinson is part of a mosaic of voices sharing tangible progress within the climate movement (Johnson & Wilkinson, 2020; Urai & Kelly, 2023). This call speaks to us as environmental and Earth scientists motivated by the urgency of climate change and social inequity, and who contribute to finding science‐driven climate solutions as part of our daily jobs. Unfortunately, we are often unable to efficiently move this critical and urgent work forward because we are impeded by cumbersome daily workflows and restrictive workplace cultures. Our workplaces have not kept pace with the modern realities of data‐intensive science: increasing data volumes and storage needs, rapidly evolving technology, new skill requirements, and a growing need for extensive and diverse collaboration. Struggling with old approaches and learning new ones in isolation can fuel burnout and turnover, preventing us from working on science‐driven climate solutions effectively.

Integrating Open Science into our work has led to indispensable scientific benefits and positive culture shifts, including expediting data workflows, improving the quality of research outputs, elevating our colleagues, building collaboration, and ultimately addressing climate change impacts. Open Science is a movement that has grown through decades of grassroots efforts and over many organizational levels (Budapest Open Access Initiative, 2001). Open Science is “the principle and practice of making research products and processes available to all, while respecting diverse cultures, maintaining security and privacy, and fostering collaborations, reproducibility, and equity” (https://open.science.gov), and the U.S. White House Office of Science and Technology Policy (OSTP) declared 2023 as a Year of Open Science that included funding and mandates to (1) strengthen policy, (2) invest in open infrastructure, (3) support the research community in building open science skills, (4) engage communities to broaden participation, and (5) promote incentives for open research practices throughout U.S. federal agencies. The Year of Open Science is helping to catalyze long‐term action, for example, with NASA making a long‐term commitment to data and computing services, data science and artificial intelligence, and Open Science implementation that includes policy, funding, education, incentives, and advocacy for Open Source software (The White House, 2024).

Here, we share our advice for connecting climate solutions to Open Science through our daily work. “Climate” need not be in our job titles or project descriptions for us to identify as part of climate solutions: all of our work considers a changing climate. We work with people and ecosystems impacted by climate change, including managing fish species that are migrating due to changing ocean temperatures, and freshwater ecosystems impacted by fluctuating drought, flooding, and pollutants. We have found vast improvements to the quality and impact of our work and our teams' morale through a culture change that helps us identify—and identifies us—as part of the Open Science and climate movements. Our experience aligns with social science research on culture change, which finds that one's knowledge, norms, attitudes, and values form a sense of self that can connect what we believe we *should* do, to our actions (Knott, 2008). Cultural change occurs through repetition and reinforcement of this sense of self (Bourdieu, 1973). In our experience, this shift started with holding intentional, respectful conversations, tapping into the knowledge base of our workplaces, and reusing effective behaviors and practices in new settings. Addressing climate change will require novel, multifaceted solutions that employ many nuanced skills and perspectives, which are best nurtured through trust and deliberate sharing of knowledge. We emphasize the need to prioritize peer learning as part of our jobs.

## 1 RAPIDLY AND RADICALLY RESHAPING CLIMATE CHANGE SCIENCE MEANS INVESTING IN BOTH TECHNICAL AND SOCIAL INFRASTRUCTURE

**FIGURE 1:**
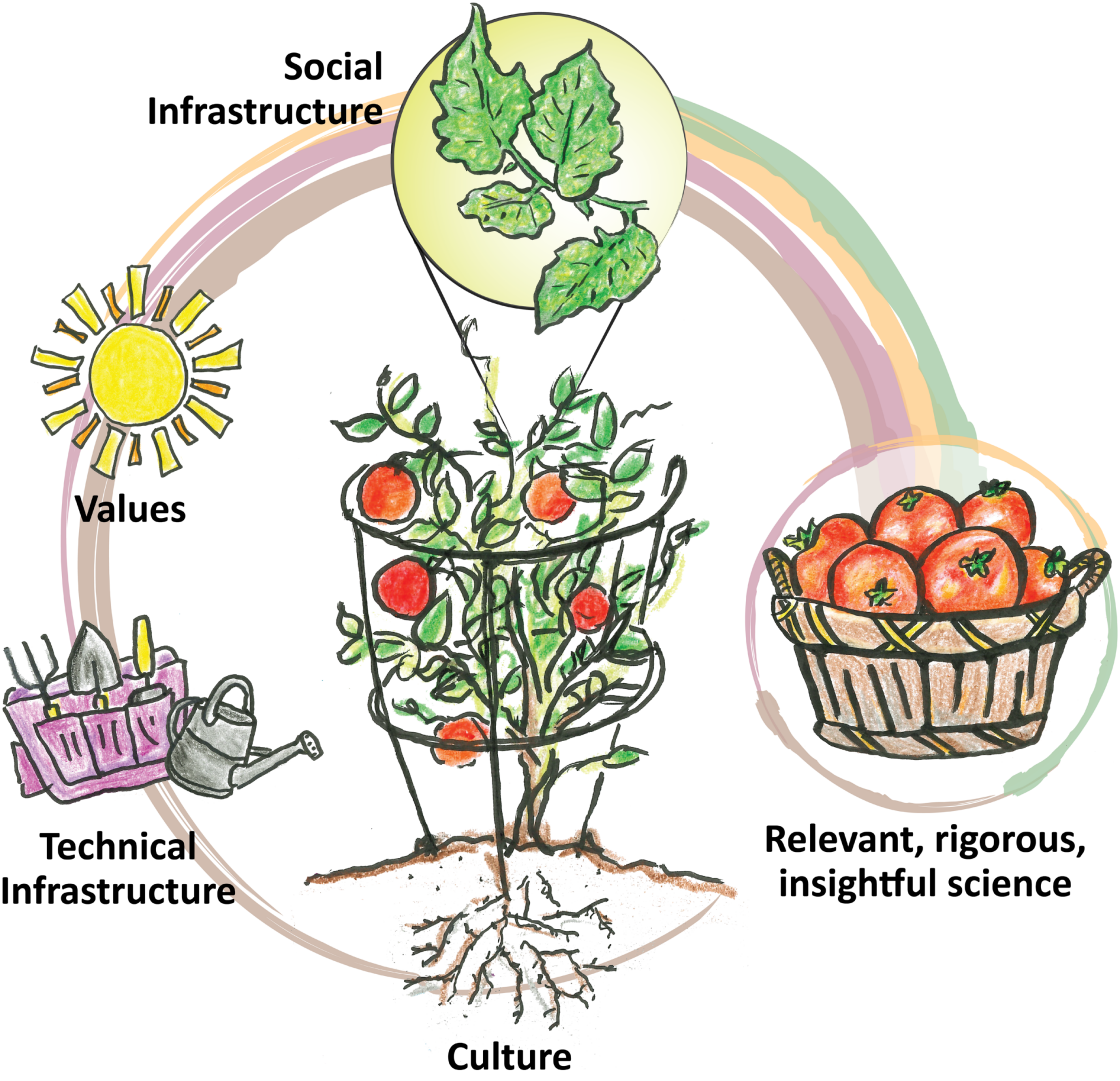
Similar to how plants use the resources available to them to develop fruits and seeds for future plants, values, culture, and technical infrastructure continually influence the generation and reproduction of relevant, rigorous, and insightful science. Illustration by Adyan Rios and Su Kim, NOAA Fisheries.

Bringing the urgency of “rapidly, radically reshaping society” (Johnson & Wilkinson, 2020) to climate‐driven Open Science requires investment not only in technical infrastructure—such as open source code (e.g., R, Python, and Julia), platforms (e.g., git, GitHub, and cloud computing), and literate programming (e.g., Jupyter Notebooks and Quarto)—but also synergistically in social infrastructure to learn these technologies and continue learning as technologies evolve (Figure 1). This is particularly true for diverse teams involved in climate change science, many of whom have not been formally trained in computing. Research teams work with increasing amounts of data and collaborators for their research and recognize the need for skills to work responsibly with data—a survey of 704 US National Science Foundation principal investigators in the biological sciences found training and teaching in data skills to be the largest unmet need (Barone et al., 2017; Williams et al., 2019, 2023). While most organizations already value the effects of “power skills” (Michail, 2022) like effective oral and written communication, action‐oriented agendas, group facilitation, and focused listening, few have truly invested in the groundwork for a supportive social infrastructure. Such social infrastructure relies on creating a culture that promotes psychological safety so that all feel welcome to speak up with diverse perspectives (Edmondson, 1999), develop growth mindsets (Dweck, 2006), and believe they have the capacity to learn (O'Keefe et al., 2018). This social infrastructure underpins healthy collaborations (Cheruvelil & Soranno, 2018) and is made possible through shared values of emotional intelligence, empathy, compassion, trust, holding space for and navigating difficult conversations, effective confrontation, and giving and receiving constructive feedback. Investing time and money in social infrastructure and explicitly acknowledging, valuing, and rewarding this otherwise invisible work helps collaborators feel comfortable sharing innovative ideas, developing new skills, asking questions, and learning from feedback, all which benefits the collective organizational culture.

Developing social infrastructure within an organization requires support from leadership and grassroots efforts (Knott, 2008). Supervisors can foster supportive social infrastructure by creating and enforcing codes of conduct, encouraging open sharing and collaboration through explicit policies, and valuing community organization and reproducible products as highly as publications (National Academies of Sciences, Engineering, and Medicine, 2021) as also occurs in Open Source software development (Braga et al., 2023; Bryan & Hester, 2022; Sholler et al., 2019). By investing in real relationships and collaboration, we can build morale through learning (Bourdieu, 1973), with individuals and teams reevaluating their objectives and workflows in light of new tools, resources, and user needs. The challenge of making substantial changes to workflows built over entire careers can be exciting for early adopters and an unsettling prospect for many others (Rogers, 2003), often exacerbated by the shame, vulnerability, and time required to learn and implement new technology (Brené Brown, 2023; Neeley, 2022). Furthermore, it can mean substantial changes to collaborating with others, challenging siloed‐work assumptions of “I work alone”, and “that person won't learn technical (or social) skills”, as well as what expertise has value, whom they can learn from, and whom they can become.

## 2 BUILDING AGENCY IN INDIVIDUALS AND MOVEMENTS ACROSS INSTITUTIONS

“Rapidly, radically reshaping” culture is about everyone having agency to take advantage of the opportunities that new technology provides. Change on a massive scale appears daunting, but social infrastructure can be built from many small opportunities and made actionable through daily habits that, together, transform how we work (Benjamin, 2022; Clear, 2019).

We offer a case study and concrete advice for building skills and confidence in ourselves and across our institutions. We have collaborated through Openscapes, an approach supporting teams with technical and social infrastructure, and Open Science (Robinson & Lowndes, 2022). We have seen and learned three main lessons by working with our teams that are connected across a bigger Open Science community. (1) We have more time to spend on science and solutions. Beyond time saved, we create better products like data‐driven reports and documentation for our research and management partners that incur less loss during day‐to‐day maintenance and succession. (2) We have improved team morale, which helps us more effectively maneuver around technical hurdles, candidly ask for help, and form truly collaborative relationships across institutions. (3) By connecting our biggest challenges around climate and social change with our daily work, we feel renewed purpose and motivation to contribute with collective agency, voice, and action.

The impact of this technical and cultural change shows up in many ways. NOAA Fisheries hosts regularly scheduled learning meetups, which during the pandemic helped teams from across the country reuse rather than reinvent similar code and onboard to the agency, an otherwise quite isolating procedure. Co‐author A. Rios estimated that “using GitHub saved me 400 would‐be emails in 4 months” because project statuses and files were easy to find and keep up to date. The US EPA Office of Research and Development and NOAA Fisheries have nascent open science communities of practice with the goal of improving skills, sharing knowledge, and supporting the adoption of open science practices. Through collaborating to support researchers using NASA Earthdata on the Cloud, the NASA Distributed Active Archive Centers (DAACs) created both conceptual cheat sheets and the *earthaccess* python library to help reduce the “time to science” (NASA Openscapes Mentors, 2022). The California Water Boards are experimenting with iterating, customizing, and scaling structured learning throughout the organization. Coauthor I. Fenwick identified a major underrepresentation of Black researchers being part of the Open Science community, and she designed a program grounded in trust and built in partnership with Black marine science faculty and community leads. The program kicked off with 80 participants and a focus on “What is Open Science anyways?” that built relationships while coworking on and screen sharing specific problems participants brought to discuss. Over time, impacts from activities like these were shared and promoted in peer‐reviewed research publications that describe R packages and workflows that others reuse in their own fisheries science (e.g., Bastille et al., 2021).

## 3 OUR BEST ADVICE: REGULARLY SCHEDULED COLLABORATIVE LEARNING MEETINGS

Our best advice to start shifting culture is to have regularly scheduled learning meetings—with structure and intention to make them different from other work meetings. We suggest naming them to give them identity and value, such as “coworking” or “Seaside Chats.” In coworking meetings, people convene in pairs or groups to seek, offer, and accept guidance from one another as they screen share and walk through a workflow, discuss a data challenge, write documentation, or learn a new tool (Lowndes et al., 2019). Coworking sessions can also be shaped around a goal or deliverable, like developing tutorials for the NASA Earthdata Cloud Cookbook. Following coworking, coauthor A. Barrett said: “I had an epiphany as to what real‐time collaborative work could be. I think it comes down to the mutual trust and respect within [coworking] that allows me to not be afraid to make mistakes or not know what I am doing, even with an audience.”

Coworking is where we learn new skills of immediate value for our daily research, from efficient keyboard shortcuts (like shift–option–arrow to highlight text on a Mac) to paired programming, an established coworking practice in software development (Fowler, 2020). It is also where we learn facilitation skills like how to design inclusive and purposeful meeting agendas that can adapt to evolving needs in real time. Coworking is where someone leans forward in their chair while a colleague is screen sharing and says “I need that. Can you teach me?” and draws from the Open Science community so that what we learn extends beyond what the team already knows. In coworking, it is critical that people opt in to being present, learning, and sharing. This helps set expectations for mutual trust and is a key element of movement building that supports the willing first (Johnson & Wilkinson, 2020; Moore, 2014).

To get started, reach out to a colleague or group that is willing and plan a time to meet. You do not have to be an expert on a given topic and there are existing lists of friendly topics to get you started (Gaynor et al., 2022; Goodman et al., 2014; Lowndes et al., 2019; Perez‐Riverol et al., 2016). Schedule a recurring slot in your calendars to help the group prioritize peer‐learning time; try weekly 1‐h or bi‐weekly 1.5‐h sessions that are amenable to time zones of the main participants, and consider accessibility and onboarding for software or physical spaces where you will convene. Create a single collaborative document (e.g., using Google Docs) for a year's worth of coworking sessions. This document gives all coworking members one place to write notes, share links, and paste screenshots that are searchable and accessible and is known as a resource if members lose internet or otherwise cannot attend (Lowndes et al., 2020).

Tell colleagues and supervisors about your coworking sessions so they can amplify and/or join. Having leaders and supervisors coworking alongside team members, and enforcing codes of conduct, can create momentum and help new practices stick across daily tasks and work environments (Lowndes et al., 2019). We see the trust and expectations established in synchronous coworking carry over into asynchronous communication spaces like Slack or Microsoft Teams and in in‐person and hybrid interactions. Making this practice visible is part of rapid and radical reshaping: it improves morale and permits other teams and organizations to adopt it as well. Coordination is more challenging but not impossible internationally; in addition to time zones and accessibility, considerations for different native spoken languages can provide a more welcoming environment.

## 4 SHIFTING INSTITUTIONAL CULTURE TO DEVELOP CLIMATE SOLUTIONS WITH OPEN SCIENCE

Through “radically, rapidly reshaping” climate change science, we are seeing cultural shifts within our institutions from one that values individual contribution to one that fosters meaningful collaboration—the clearest path to the innovations necessary to mitigate the impending climate change‐related shocks. This work is hard and messy, and progress is not linear; it is only possible if all participants feel comfortable asking questions about what they do not understand and are received with patience and interest (Lowndes, 2019). It is not about ephemeral alliances to avoid conflict, rather it is about long‐term collaboration (e.g., giving and receiving feedback and fostering a reflection and growth mindset) where individuals have the toolsets to face new and/or hard problems (Robinson & Lowndes, 2022).

Support and enthusiasm from leadership to connect developing new skills with technology‐enabled culture change is critical. This is a big time investment, and we need every solution and every solver (Johnson & Wilkinson, 2020). We also need to start from a coalition of the willing; recognizing where our energy is best spent and learning from other Open Science groups rather than reinventing. This includes identifying shared challenges that bring different groups like early adopters and leadership together (Moore, 2014); for example making annual reports less manually time‐intensive and error‐prone have been a shared motivator connecting many groups across NOAA Fisheries. Shifting culture also involves continually encouraging and welcoming others by pointing to other peer examples of groups shifting to Open Science (Lowndes et al., 2019; Robinson & Lowndes, 2022) and rewarding time spent in employee performance plans and promotion, as well as policy mandates that affect the way we all work (Nelson, 2022; The White House, 2024). Finding solutions to climate change necessitates bringing together diverse talent, sharing knowledge, using the latest technological advances, and supporting effective teams. This means that no matter the role you play, you have a place in the Open Science movement: please join us.

## AUTHOR CONTRIBUTIONS

**Julia Stewart Lowndes:** Conceptualization (equal); funding acquisition (equal); methodology (equal); writing – original draft (equal); writing – review and editing (equal). **Anna M. Holder:** Conceptualization (equal); methodology (equal); writing – original draft (equal); writing – review and editing (equal). **Emily H. Markowitz:** Conceptualization (equal); methodology (equal); writing – original draft (equal); writing – review and editing (equal). **Corey Clatterbuck:** Conceptualization (equal); methodology (equal); writing – original draft (equal); writing – review and editing (equal). **Amanda L. Bradford:** Conceptualization (equal); methodology (equal); writing – original draft (equal); writing – review and editing (equal). **Kathryn Doering:** Conceptualization (equal); methodology (equal); writing – original draft (equal); writing – review and editing (equal). **Molly H. Stevens:** Conceptualization (equal); methodology (equal); writing – original draft (equal); writing – review and editing (equal). **Stefanie Butland:** Conceptualization (equal); methodology (equal); writing – original draft (equal); writing – review and editing (equal). **Devan Burke:** Conceptualization (equal); methodology (equal); writing – original draft (equal); writing – review and editing (equal). **Sean Kross:** Conceptualization (equal); methodology (equal); writing – original draft (equal); writing – review and editing (equal). **Jeffrey W. Hollister:** Conceptualization (equal); methodology (equal); writing – original draft (equal); writing – review and editing (equal). **Christine Stawitz:** Conceptualization (equal); methodology (equal); writing – original draft (equal); writing – review and editing (equal). **Margaret C. Siple:** Conceptualization (equal); methodology (equal); writing – original draft (equal); writing – review and editing (equal). **Adyan Rios:** Conceptualization (equal); methodology (equal); writing – original draft (equal); writing – review and editing (equal). **Jessica Nicole Welch:** Conceptualization (equal); methodology (equal); writing – original draft (equal); writing – review and editing (equal). **Bai Li:** Conceptualization (equal); methodology (equal); writing – original draft (equal); writing – review and editing (equal). **Farnaz Nojavan:** Conceptualization (equal); methodology (equal); writing – original draft (equal); writing – review and editing (equal). **Alexandra Davis:** Conceptualization (equal); methodology (equal); writing – original draft (equal); writing – review and editing (equal). **Erin Steiner:** Conceptualization (equal); methodology (equal); writing – original draft (equal); writing – review and editing (equal). **Josh M. London:** Conceptualization (equal); methodology (equal); writing – original draft (equal); writing – review and editing (equal). **Ileana Fenwick:** Conceptualization (equal); methodology (equal); writing – original draft (equal); writing – review and editing (equal). **Alexis Hunzinger:** Conceptualization (equal); methodology (equal); writing – original draft (equal); writing – review and editing (equal). **Juliette Verstaen:** Conceptualization (equal); methodology (equal); writing – original draft (equal); writing – review and editing (equal). **Elizabeth Holmes:** Conceptualization (equal); methodology (equal); writing – original draft (equal); writing – review and editing (equal). **Makhan Virdi:** Conceptualization (equal); methodology (equal); writing – original draft (equal); writing – review and editing (equal). **Andrew P. Barrett:** Conceptualization (equal); methodology (equal); writing – original draft (equal); writing – review and editing (equal). **Erin Robinson:** Conceptualization (equal); funding acquisition (equal); methodology (equal); writing – original draft (equal); writing – review and editing (equal).

## FUNDING INFORMATION

This work was funded in part by NASA ROSES 80NSSC21K0564 Award # 20‐TWSC20‐2‐0003 (JSS Lowndes & EM Robinson).

## CONFLICT OF INTEREST STATEMENT

Authors declare no conflict of interest.

## DISCLAIMERS

The views expressed in this article are those of the authors and do not necessarily represent those of the California State Water Resources Control Board.

The views expressed in this article are those of the authors and do not necessarily represent the views or policies of the U.S. Environmental Protection Agency. Any mention of trade names, products, or services does not imply an endorsement by the U.S. Government or the U.S. Environmental Protection Agency. The EPA does not endorse any commercial products, services, or enterprises.

The scientific results and conclusions, as well as any views or opinions expressed herein, are those of the author(s) and do not necessarily reflect those of NOAA or the Department of Commerce.

The scientific results and conclusions, as well as any views or opinions expressed herein, are those of the author(s) and do not necessarily reflect those of USGS or the Department of the Interior.

Exhibit: Notice of Copyright.

Notice: This manuscript has been authored by UT‐Battelle, LLC, under contract DE‐AC05‐00OR22725 with the US Department of Energy (DOE). The US government retains and the publisher, by accepting the article for publication, acknowledges that the US government retains a non‐exclusive, paid‐up, irrevocable, worldwide license to publish or reproduce the published form of this manuscript, or allow others to do so, for US government purposes. DOE will provide public access to these results of federally sponsored research in accordance with the DOE Public Access Plan (https://www.energy.gov/doe‐public‐access‐plan).

## Data Availability

No data were analyzed for this article. Preprint available at: https://eartharxiv.org/repository/view/5948/.
